# Predicting the response to neoadjuvant chemotherapy for breast cancer: wavelet transforming radiomics in MRI

**DOI:** 10.1186/s12885-020-6523-2

**Published:** 2020-02-05

**Authors:** Jiali Zhou, Jinghui Lu, Chen Gao, Jingjing Zeng, Changyu Zhou, Xiaobo Lai, Wenli Cai, Maosheng Xu

**Affiliations:** 1 Department of Radiology, The First Affiliated Hospital of Zhejiang Chinese Medical University, 54 Youdian Road, Shangcheng District, Hangzhou, 310006 People’s Republic of China; 2 The First Clinical Medical College of Zhejiang Chinese Medical University, Hangzhou, China; 3 Ningbo First Hospital, Ningbo, China; 4 Department of Radiology, Massachusetts General Hospital and Harvard Medical School, 25 New Chardon St., 400C, Boston, MA 02114 USA; 5 The Third Affiliated Hospital of Zhejiang Chinese Medical University, Hangzhou, China

**Keywords:** Radiomics, Breast cancer, Neoadjuvant chemotherapy, Pathological complete response

## Abstract

**Background:**

The purpose of this study was to investigate the value of wavelet-transformed radiomic MRI in predicting the pathological complete response (pCR) to neoadjuvant chemotherapy (NAC) for patients with locally advanced breast cancer (LABC).

**Methods:**

Fifty-five female patients with LABC who underwent contrast-enhanced MRI (CE-MRI) examination prior to NAC were collected for the retrospective study. According to the pathological assessment after NAC, patient responses to NAC were categorized into pCR and non-pCR. Three groups of radiomic textures were calculated in the segmented lesions, including (1) volumetric textures, (2) peripheral textures, and (3) wavelet-transformed textures. Six models for the prediction of pCR were Model I: group (1), Model II: group (1) + (2), Model III: group (3), Model IV: group (1) + (3), Model V: group (2) + (3), and Model VI: group (1) + (2) + (3). The performance of predicting models was compared using the area under the receiver operating characteristic (ROC) curves (AUC).

**Results:**

The AUCs of the six models for the prediction of pCR were 0.816 ± 0.033 (Model I), 0.823 ± 0.020 (Model II), 0.888 ± 0.025 (Model III), 0.876 ± 0.015 (Model IV), 0.885 ± 0.030 (Model V), and 0.874 ± 0.019 (Model VI). The performance of four models with wavelet-transformed textures (Models III, IV, V, and VI) was significantly better than those without wavelet-transformed textures (Model I and II). In addition, the inclusion of volumetric textures or peripheral textures or both did not result in any improvements in performance.

**Conclusions:**

Wavelet-transformed textures outperformed volumetric and/or peripheral textures in the radiomic MRI prediction of pCR to NAC for patients with LABC, which can potentially serve as a surrogate biomarker for the prediction of the response of LABC to NAC.

## Background

Breast cancer is the most common malignant tumor among women across the world [1, 2]. For treatment, preoperative neoadjuvant chemotherapy (NAC) plays a major role in patients with locally advanced breast cancer (LABC) [3]. With proper therapy, NAC has been shown to decrease tumor size, downstage tumors, and allow breast-conserving surgery to take place with clearer margins [4]. Furthermore, timely NAC therapy can also improve the efficacy of follow-up treatment options after surgery [5].

The response of breast cancer to NAC relies on the post-treatment pathology, and the pathological complete response (pCR) is clinically defined as having no residual invasive carcinoma in the breast tissue after surgery, which is associated with a better prognosis [6]. However, it has been reported that the pCR rate of NAC for breast cancer varies between 10 and 50% [7]. This poor pCR rate signifies that the majority of patients receiving NAC may benefit from a treatment course other than NAC. Given the well-documented adverse effects to chemotherapy [8], an urgent clinical need is present for objective surrogate biomarkers to accurately predict the response of breast cancer to NAC.

Radiomics is an emerging technology in quantitative imaging analysis, which hypothesizes that the spatial tumor heterogeneity is related to tissue changes on histological analysis. Preliminary studies using radiomics for breast MRI have shown that certain pre-treatment texture parameters (based on high order statistics) may help in evaluating breast tumor response to NAC [9–12]. Previously, high throughput image textures have been obtained for radiomics analysis to predict the efficacy of NAC prior to initiating treatment [13, 14]. The majority of studies applied morphologic features and gray-level textures (such as histogram, gray-level co-occurrence matrices, etc) extracted from regions of interest (ROI). Alternatively, wavelet transformation can provide comprehensive spatial, and frequency distributions for characterizing intratumoral and peritumoral regions in terms of low and high frequency signals. These properties may improve the performance of radiomic model [15, 16]. The aim of this study was to investigate whether wavelet-transformed textures can improve the performance of radiomic MRI predictions of pCR to NAC in comparison to those utilizing various combinations of volumetric textures, peripheral textures, and wavelet-transformed textures extracted in breast MRI.

## Methods

### Patients

The Ethics Committee of the First Affiliated Hospital of Zhejiang Chinese Medical University has approved this retrospective study, in which informed consent was waived, but patient confidentiality was protected. All patients with LABC who received NAC were collected in our institution from January 2013 to December 2017. Inclusion criteria for this study were: (1) An adult female patient over 18 years old; (2) Puncture biopsy confirmed unilateral invasive ductal carcinoma of the breast prior to NAC therapy; and (3) CE-MRI examination was performed within 2 weeks before NAC.

The exclusion criteria were as follows: (1) Patients who underwent the aspiration biopsy or accepted any endocrine or radiation therapy before MRI scans; (2) The baseline CE-MRI scan was performed more than 1 week before NAC; (3) Lesions were scarcely identified on MRI due to motion or other artifacts; (4) Neoadjuvant chemotherapy was not completed due to extraneous reasons; (5) Patients who did not perform surgical resection after the completion of NAC.

**Fig. 1:**
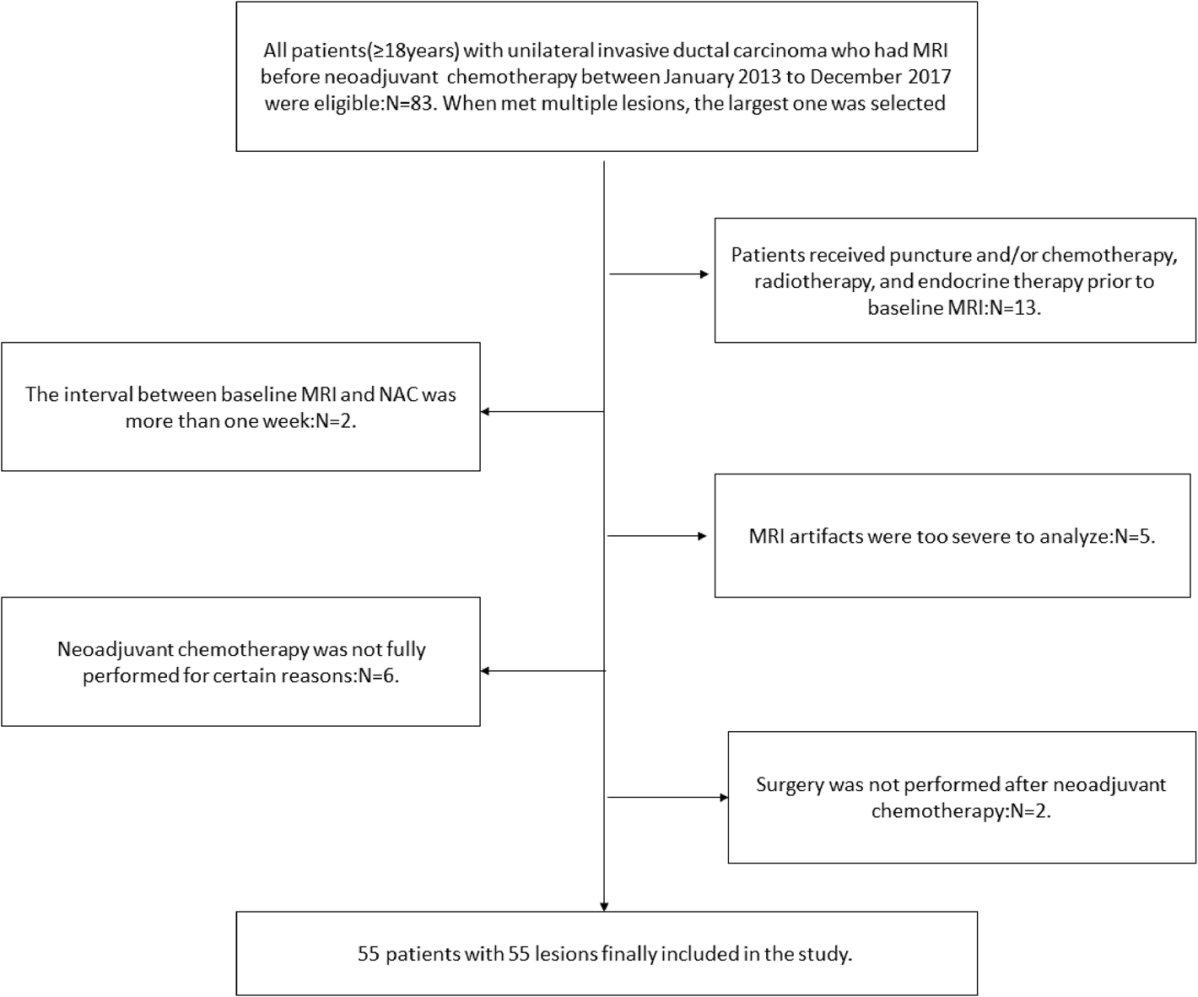
Flow diagram of the patient selection in the study

Figure 1 shows the identification, eligibility, and inclusion of patients in the study. A total of 55 patients were selected from initial identification of 83 patients after exclusion of patients who underwent other therapies before MRI (*n* = 13), those where more than 1 week had passed between MRI and NAC (*n* = 2), those with severe image artifacts (*n* = 5), incomplete NAC (*n* = 6), and those with no surgical resection after NAC (*n* = 2).

### Clinical and pathological data

The patient’s clinical data including patient’s age, tumor size, tumor histopathologic type, molecular subtypes, lymph node invasion before NAC, tumor types (mass vs non-mass) in CE-MRI, and the regimen of NAC were retrieved from the hospital’s medical record system. The pathological assessment of NAC was acquired from the pathology reports after breast-conserving surgery or mastectomy, which was completed by a pathologist with more than 10 years of working experience. The criteria of pCR were defined as the absence of residual invasive tumor in the surgical specimen (residual ductal carcinoma in situ could be present) and the absence of lymph node invasion in the ipsilateral sentinel node or lymph nodes removed during the axillary dissection.

### Image acquisition

Each patient underwent DCE-MRI examination on a 3.0 Telsa MR scanner (Siemens, Erlangen, Germany) in the prone position with the use of a dedicated 16-channel bilateral phased-array breast coil for signal reception. Data was obtained for routine clinical practice. The DCE-MRI imaging protocol was as follows: TR/TE = 4.51 ms /1.61 ms, section thickness, 1 mm; flip angle, 10°. The matrix was 448 × 448; and NEX = 6). One non-contrast and five contrast dynamic series were included into the DCE-MRI imaging. The fifth phase of imaging was selected into segmentation at 245 s after contrast injection. The gadolinium chelate was injected via the basilic vein with the dosage of 0.1 mmol/kg body weight, followed by a 10 mL flush of isotonic saline.

### Tumor segmentation

Each tumor was segmented on enhanced T1-weighted images using a semi-automated segmentation tool in an open volumetric image analysis platform 3DQI (an open software platform for volumetric image analysis developed by the 3D quantitative imaging laboratory at Massachusetts General Hospital and Harvard Medical School (https://3dqi.mgh.harvard.edu), focusing on the prediction and assessment of the treatment response in clinical oncology). Each tumor was first identified and segmented on the axial plane by a breast radiologist with 3 years’ experience and then verified by another breast radiologist with 7 years’ experience. They were blinded to the pathological assessment of NAC after surgery. The corresponding sagittal and coronal planes of the tumor were referenced when the lesion was ambiguous in the axial plane. The volumes of interest (VOIs) of each tumor was determined by the consensus of both radiologists. In the case of multiple lesions in a patient, the largest detected lesion was selected.

### Radiomic analysis

3DQI software (3D Quantitative Imaging Lab, Harvard Medical School) was utilized to texture calculation and radiomic analysis. Three groups of radiomic textures were calculated for the segmented lesions, including volumetric, peripheral textures, and wavelet-transformed textures. Volumetric textures were calculated in the entire volume of segmented lesion containing 5 categories: 11 shape features, 25 histogram statistical textures, 22 gray level co-occurrence matrix (GLCM) textures, 16 gray level run-length matrix (GLRLM) textures, and 14 gray level zone size matrix (GLZSM) textures. Peripheral textures were calculated in a 10 mm wide band region centered on the boundary of the segmented lesions, which covered the 5 mm inner region and 5 mm outer region separated by the lesion boundary. We calculated 77 volumetric textures except 11 shape features in the periphery region.

A 3D discrete and single-stage wavelet transform was used to decompose volumetric images into eight decomposed volumes of images, labeled as LLL, LLH, LHL, LHH, HLL, HLH, HHL and HHH, where L and H are low- and high-frequency signals, respectively. For example, LLH is a volume of images transformed by using the low-pass filters on the X and Y axis, and a Z-axis high-pass filter. In the eight decomposed volumes of images, 3DQI calculated five categories of volumetric textures with the exception of the shape features in the segmented lesion VOIs, which resulted in a total of 616 (8X77) wavelet-transformed texture features for each VOI.

A random forest (RF) was applied [17] to predict the response of pCR to NAC using tumor texture features calculated from the pre-operative CE-MRI. RF is a machine learning classifier, which can prevent over-fitting of the data (due to a large number of radiomic features) by injecting randomness into the training of the trees and combining the output of multiple random trees into the final classifier. Thus, a random forest is known to perform consistently well in high-dimensional data compared with other classification algorithms [17]. We trained six RF models to the prediction of pCR by using six combinations of three groups of radiomic textures along with the clinical outcomes. Each RF classification model had 100 trees with a node size of 1. The number of features for each tree is the square root of the total number of features in each model (rounded up). To avoid over-fitting, RF randomly chooses a subset of features (feature bagging) with respect to the number of features to grow each tree, and randomly sampled the subset of the bootstrapped data (sample bagging). The six radiomics combinations were Model I: volumetric textures, Model II: volumetric+peripheral textures, Model III: wavelet textures, Model IV: volumetric+wavelet textures, Model V: peripheral+wavelet textures, and Model VI: volumetric+peripheral+wavelet textures.

For the selection of important textures in each model, we adopted a two-round feature selection scheme to select the optimal features for each model. First, the importance scores calculated by the Boruta algorithm were used for a rapid reduction of texture dimensionality [18]. The Boruta algorithm is a feature ranking and selection algorithm based on the random forests algorithm, which identifies all features which are either strongly or weakly relevant to the decision variable. The importance of a feature is defined by the loss of classification accuracy caused by the random permutation of feature values between objects. Non-relevant features were rejected by using Z score cutoff of less than 0.01. An initial RF model was established after the first round by including all relevant features. At the second round, an iterative culling-out algorithm was used to refine the model [19]. In each iteration, we calculated the prediction performance of the RF model by removing one of the textures, i.e. the AUC value of the ROC curve. If the AUC value using one-less texture parameter is higher than that of current RF model, the model corresponding to the maximum AUC value was selected. This iteration was completed until no AUC values ​​were higher than that of the current model.

**Fig. 2:**
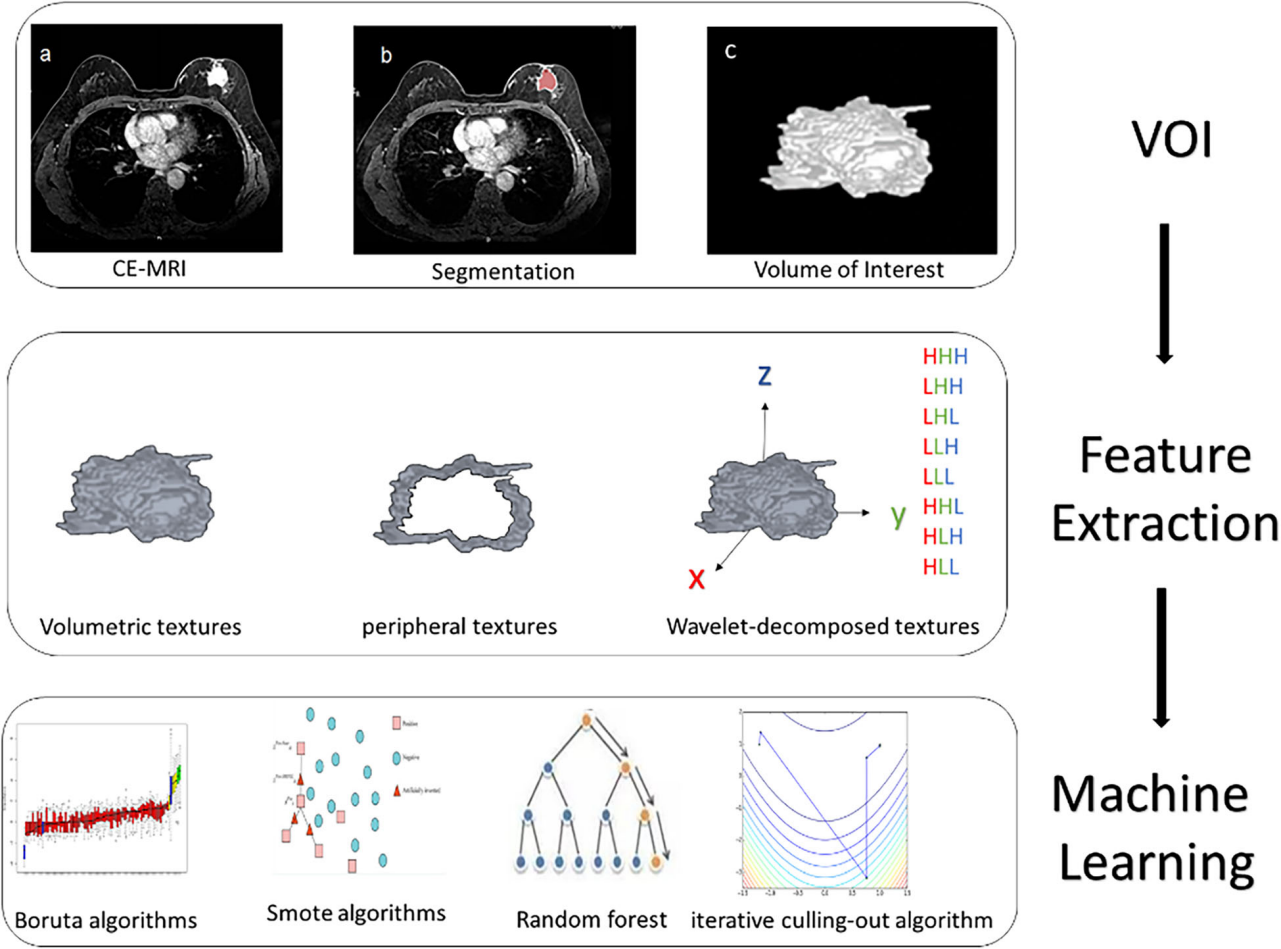
Radiomic MRI prediction of pathological complete response (pCR)

To reduce the bias that may be caused by an unbalanced number of positive and negative samples, we applied the SMOTE (Synthetic Minority Oversampling Technique) resampling method [20, 21], which combines informed oversampling of the minority class (patients with small number of tumors) with random undersampling of the majority class (patients with large number of tumors) to balance the samples between different patient groups. All radiomic features in each patient group were resampled to 50 tumor radiomic samples by SMOTE method, which resulted in 100 samples including 50 pCR and 50 nonpCR samples. A 10-fold cross-validation method was applied to train and validate the model through *n* = 100 repetitions. The model performance was compared by using the AUC values, represented by mean ± SD. Figure 2 shows the pipeline of our RF models for prediction of pCR.

### Statistical analysis

All statistical analyses were performed in SPSS version 19.0. A Chi square test or Fisher’s exact test was used for the nominal variable. A Mann-Whitney U test was used for the unordered categorical variable. A student’s t test was used for the continuous variable. A *p*-value less than 0.05 was considered statistically significant.

## Results

### Clinical and pathological data

**Fig. 3:**
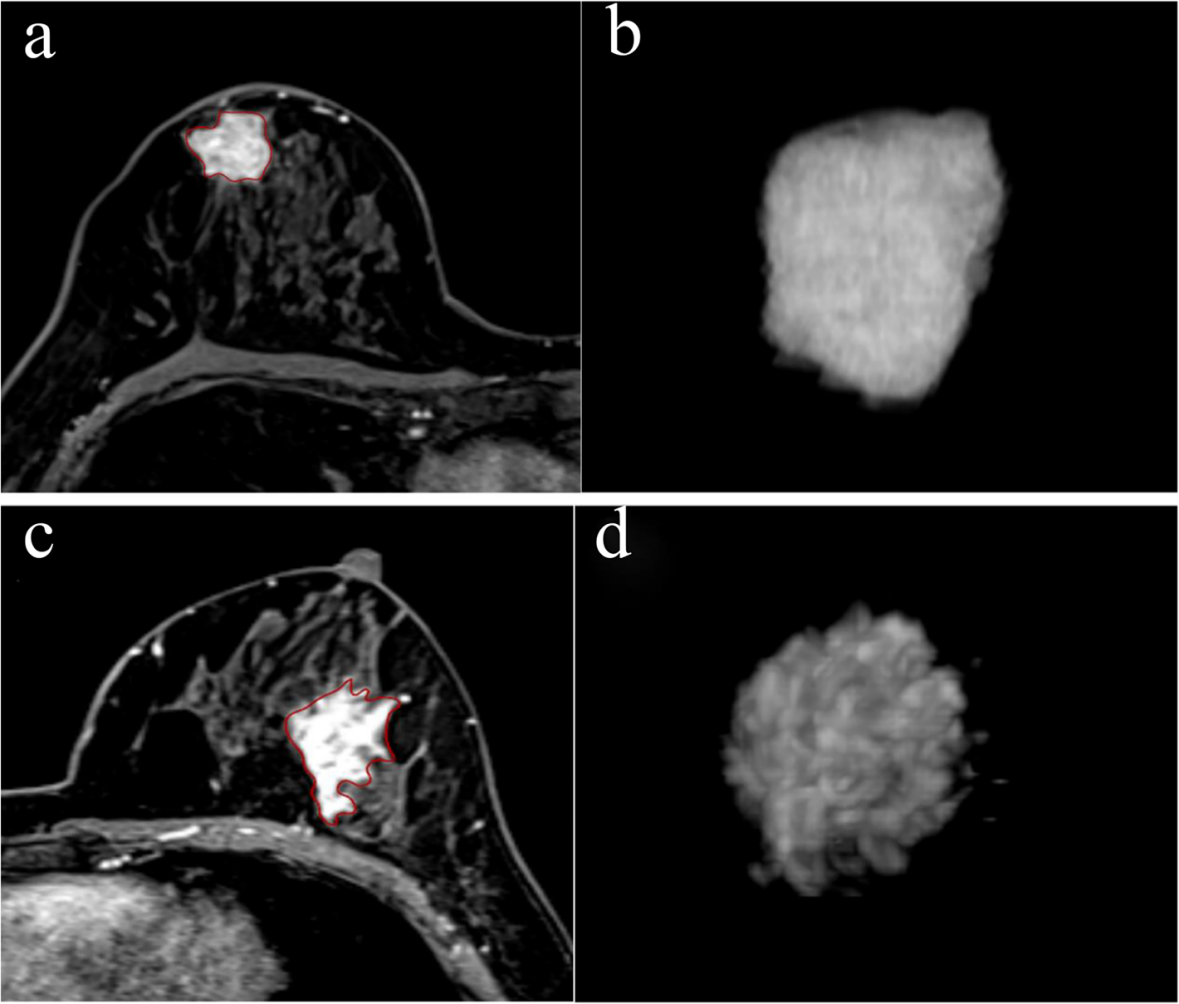
Segmentation of breast lesions on CE-MRI. Images **a**-**b** show the right invasive breast cancer that was non-pCR after NAC. Images c-d show the left invasive breast cancer that was pCR after NAC. **a**, **c** Segmentation of breast lesions on CE-MRI. **b**, **d** 3D imaging of VOIs

Fifty-five female patients aged 25 to 75 years (mean age = 50.4 ± 12.2 years) were enrolled in the study which included 49 patients with a single breast tumor and 6 patients with multiple tumors. All patients were diagnosed with invasive ductal carcinoma by pre-NAC puncture biopsy and received NAC prior to surgical resection. Clinical and pathological data of the study were listed in Table 1. The pCR rate was 30.9% (17/55) (mean age = 50.7 ± 9.4 years), whereas non-pCR rate was 69.1% (38/55) (mean age = 49.5 ± 10.4 years). The median maximum diameters of the lesions were 2.6 cm (range: 2.3–3.7 cm) and 4.2 cm (range 3.1–5.4 cm) in the pCR and the non-pCR group, respectively; and the mean diameters were 2.9 ± 1.1 cm and 4.3 ± 1.9 cm, respectively. Except for the maximum diameter (*p* = 0.002), there were no statistically significant differences between pCR and non-pCR groups of patients. Figure 3 demonstrates the segmentation of breast lesions on CE-MRI.

**Table 1:** Clinical and pathological data in the study

	pCR	Non-pCR	P-value
No. of patients	17	38	N/A
Age(y)			
Median(range)	50 (37–70)	48 (25–68)	N/A
Mean ± SD	50.7 ± 9.4	49.5 ± 10.4	0.676
Enhancement Type, No. (%)			0.506
Masslike	11 (64.7)	23 (60.5)	
Non-masslike	6 (35.3)	15 (39.5)	
Max-D(cm)*			
Median(range)	2.6 (2.3–3.7)	4.2 (3.1–5.4)	N/A
Mean ± SD	2.9 ± 1.1	4.3 ± 1.9	0.002
Subtype, No. (%)			0.493
Luminal A	5 (29.4)	17 (44.7)	
Luminal B	2 (11.8)	7 (18.4)	
Her-2	5 (29.4)	8 (21.1)	
TNBC	5 (29.4)	6 (15.8)	
Regimen, No. (%)			0.412
EC + Taxol	4 (23.5)	14 (36.8)	
FEC + Taxol	7 (41.2)	15 (39.5)	
AC+ Taxol	2 (11.8)	6 (15.8)	
Others	4 (23.5)	3 (7.9)	

NOTE. P-values were calculated by T-test or Mann-Whitney U test for Age, Max-D, from Chi-square test or Fisher’s exact test for Enhancement type, Subtype, Regimen

Abbreviations: *Max-D* Maximum- diameter, *Her-2* Human epidermal growth factor receptor 2, *TNBC* Triple negative breast cancer, *E* epirubicin; *C* cyclophosphamide, *Taxol* paclitaxel; *F* 5-fluoroucil. *A* doxorubicin. *N/A* Not available

### Radiomic models

**Fig. 4:**
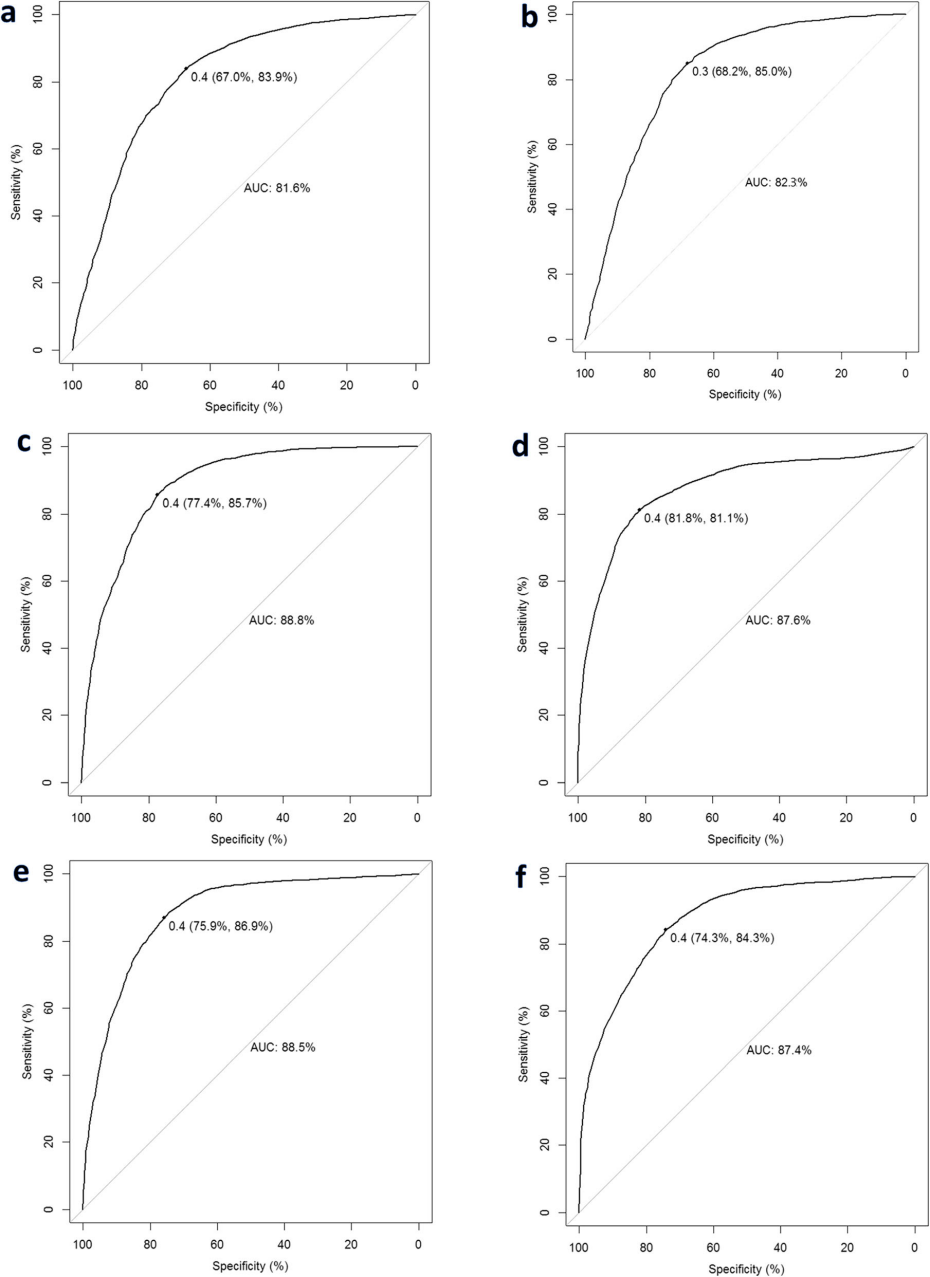
Receiver operating characteristic (ROC) curves of the six RF models: **a** Model I: volumetric textures, **b** Model II: volumetric + peripheral textures, **c** Model III: wavelet textures, **d** Model IV: volumetric + wavelet textures, **e** Model V: peripheral + wavelet textures, and **f** Model VI: volumetric + peripheral + wavelet textures

After applying our feature selection method to three groups of MRI radiomic textures, we identified 5 volumetric texture features, 3 peripheral texture features, and 3 wavelet texture features, respectively, for the prediction of pCR. Table 2 lists the six models by their combinations of the three groups of selected features. The AUCs of the six models for the prediction of pCR were 0.816 ± 0.033 (Model I: volumetric textures), 0.823 ± 0.020 (Model II: volumetric + peripheral textures), 0.888 ± 0.025 (Model III: wavelet textures), 0.876 ± 0.015 (Model IV: volumetric + wavelet textures), 0.885 ± 0.030 (Model V: peripheral + wavelet textures), and 0.874 ± 0.019 (Model VI: volumetric + peripheral + wavelet textures). Figure 4 shows the ROCs and AUC values of the six models.

**Table 2:** Textures and performance (AUC, Accuracy, Sensitivity and Specificity) of six RF models

	RF Models					
	I	II	III	IV	V	VI
Features	Volumetric	Volumetric + Peripheral	Wavelet	Volumetric + Wavelet	Peripheral + Wavelet	Volumetric + Peripheral + Wavelet
Selected Features	GLZSM_salgle GLCM_homo1 GLCM_diffEntro GLCM_dissimilar SHAPE_surfaceArea	GLZSM_salgle GLCM_homo1 GLCM_diffEntro GLCM_dissimilar Bndry_RL_rln Bndry_GLZSM_salgle Bndry_GLCM_contrast SHAPE_surfaceArea	LHH_GLZSM_zp LLH_GLCM_infoCorr1 HHH_GLCM_correlation	GLZSM_salgle GLCM_homo1 GLCM_diffEntro GLCM_dissimilar HHH_GLCM_correlation LHH_GLZSM_zp LLH_GLCM_infoCorr1 SHAPE_surfaceArea	Bndry_RL_rln Bndry_GLZSM_salgle Bndry_GLCM_contrast HHH_GLCM_correlation LHH_GLZSM_zp LLH_GLCM_infoCorr1	SHAPE_surfaceArea GLZSM_salgle GLCM_homo1 GLCM_diffEntro GLCM_dissimilar Bndry_RL_rln Bndry_GLZSM_salgle Bndry_GLCM_contrast HHH_GLCM_correlation LHH_GLZSM_zp LLH_GLCM_infoCorr1
AUC (mean ± SD)	0.816 ± 0.033	0.823 ± 0.020	0.888 ± 0.025	0.876 ± 0.015	0.885 ± 0.030	0.874 ± 0.019
Accuracy (mean ± SD)	0.747 ± 0.022	0.751 ± 0.0150	0.810 ± 0.030	0.781 ± 0.028	0.797 ± 0.032	0.787 ± 0.024
Sensitivity (mean ± SD)	0.676 ± 0.043	0.684 ± 0.043	0.762 ± 0.035	0.730 ± 0.049	0.770 ± 0.034	0.727 ± 0.045
Specificity (mean ± SD)	0.812 ± 0.023	0.798 ± 0.021	0.845 ± 0.031	0.818 ± 0.037	0.818 ± 0.047	0.830 ± 0.035

NOTE: Bndry_GLZSM_salgle, Bndry_GLCM_contrast and Bndry_RL_rln were peripheral texture features

**Fig. 5:**
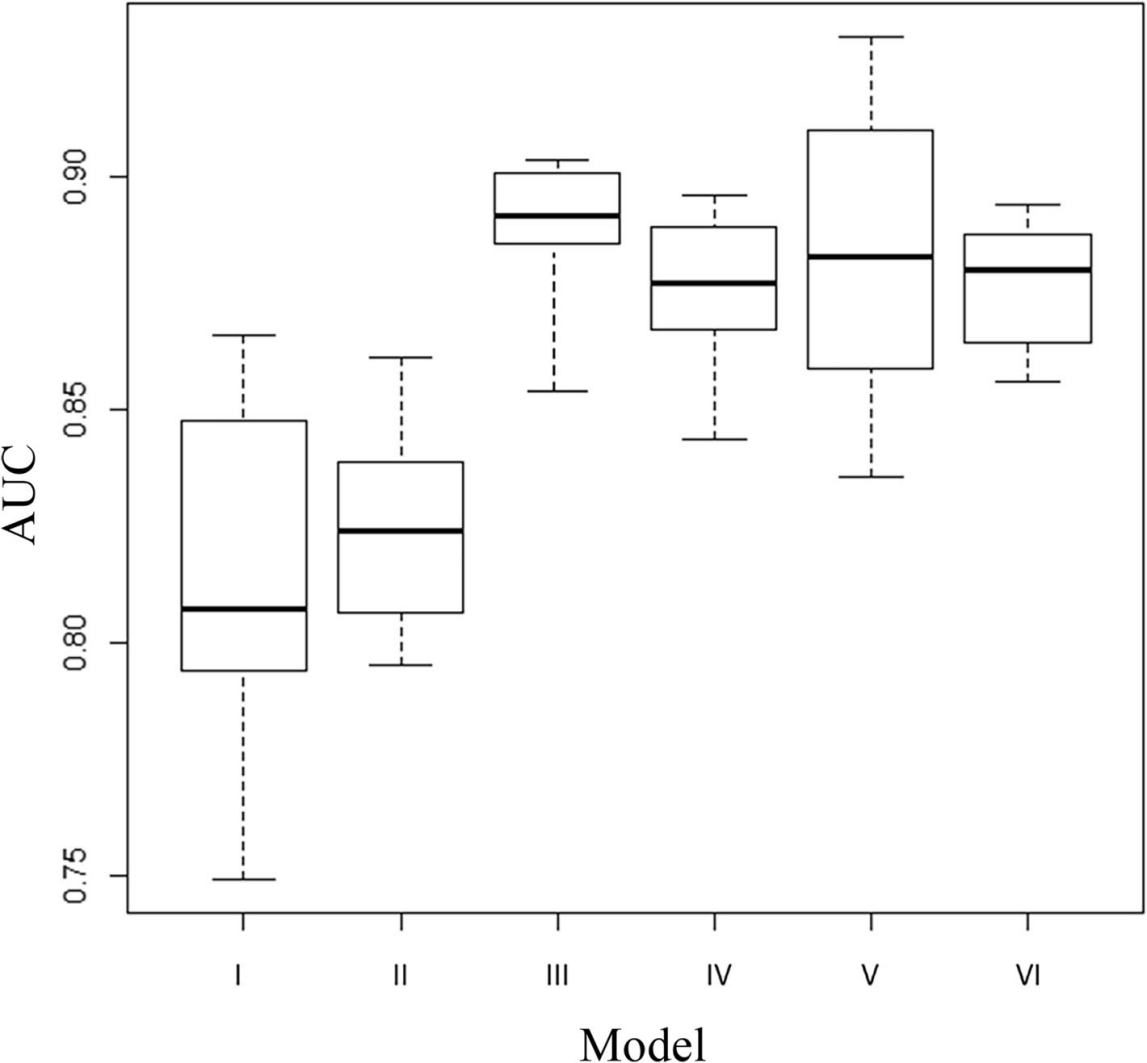
Boxplots of the prediction performance (AUC, area under ROC curve) of six radiomics models

The performance (AUC, accuracy, sensitivity, and specificity) of four models with wavelet textures (Models III, IV, V, and IV) were statistically significantly better than those without wavelet textures (Model I and II). The models by inclusion of peripheral textures did not show significant improvements in performance compared to those exclusion of peripheral textures (Model I vs Model II, *p* = 0.985; Model III vs Model V, *p* = 1.000). Also, the addition of either volumetric textures or peripheral textures or both to the wavelet textures (Models IV vs Model III, *p* = 0.891; Model V vs Model III, *p* = 1.000; Model VI vs Model III, *p* = 0.809) did not yield any improvements in performance compared to the model with wavelet textures only (Model III). Figure 5 plots the AUCs of the six models, and Table 3 lists the *p*-values among the six models.

**Table 3:** Comparison of p-values of AUCs between 6 models

Models	I	II	III	IV	V	VI
I	N/A	0.985	< 0.001	< 0.001	< 0.001	< 0.001
II		N/A	< 0.001	< 0.001	< 0.001	0.001
III			N/A	0.891	1.000	0.809
IV				N/A	0.968	1.000
V					N/A	0.924
VI						N/A

NOTE: P-values are calculated from the T-test

## Discussions

Several studies have investigated radiomics models in the prediction of the response of breast cancer to NAC on CE-MRI [10–12, 22], as shown in Additional file 1: Table S1. However, the performance of these models varied due to the different types of radiomic features extracted and different types of VOIs applied, intratumoral or peritumoral regions. This study investigated six radiomics models composed of three groups of textures, volumetric textures, peripheral textures, and wavelet-transformed textures, for prediction of pCR to NAC in breast CE-MRI.

Among 88 volumetric textures, five features were selected as important features, of which three textures were chosen from GLCM features. Prior studies have demonstrated that GLCM may be associated with intratumoral heterogeneity, and high intratumoral heterogeneity may be associated with poor prognosis [23, 24]. GLCM features were also applied to predict chemotherapy response to triple negative breast cancer [22], which was consistent with the results of our study. In addition, tumor shape features may improve the prediction of prognosis of LABC underwent NAC [25]. For instance, tumor size and tumor surface characteristics were related to the effectiveness of NAC [26, 27]. In the group of peripheral texture features, three features were selected, one from each of the GLCM, GLZSM, and RL families, respectively, representing the homogeneity and heterogeneity of peripheral tumor regions [9, 28]. The wavelet transformation decomposes images into high frequency (heterogeneity) and low frequency (homogeneity) for both intratumoral and peritumoral regions [29]. The HHH_GLCM family highlighted the features of the tumor boundary and any internal inhomogeneity. The LLH_GLCM family revealed the intra-slice homogeneity and inter-slice inhomogeneity characterization. Peritumoral textures may be characterized by the high frequency signals in the tumor boundary regions in the wavelet-transformed images [11], whereas, intratumoral textures may be characterized by the low frequency domains in wavelet-decomposed images.

In our study, four models with wavelet-transformed textures (Model III to VI) outperformed Model I and II without wavelet textures in the prediction of pCR to NAC of LABC (*p* <  0.001). This indicated that the inclusion of wavelet-transformed features may improve the performance of the prediction models, which is consistent with the results of Imon Banerjee et al. [30] In general, lesion edges are related to the high-frequency signals in the wavelet-transformed images. Prior studies have demonstrated that the addition of peritumoral texture features optimized the performance for predicting pCR of NAC [11]. In our study, Model II achieved a better performance than Model I after the addition of peritumoral texture features, but without statistical significance (*p* = 0.892). On the other hand, in the comparison of Models III to VI, we observed that the inclusion of the peritumoral texture features into the wavelet-decomposed textures (Model VI) did not show significant improvements in the model (Model III). This revealed that characterization from intertumoral and peritumoral textures may be contained in the wavelet-decomposed textures. The wavelet-transformed textures achieved the best performance for radiomic MRI prediction of the pCR of NAC for breast cancer. Thus, wavelet-transformed textures may be sufficient to predict pCR of NAC without calculation of textures separately in the intertumoral and peritumoral regions.

A 3D discrete wavelet transformation decomposes images into one approximation and seven detailed images, which are mutually orthogonal sets of wavelets, representing the low-frequency (smooth such as homogeneous intertumoral region) and high-frequency (non-smooth such as tumor boundaries or heterogeneous intertumoral region) contents of the images, respectively, which are not affected by motion or orientation. On the other hand, Gabor wavelets are claimed to be sensitive for detecting the local texture features corresponding to specific orientations, allowing optimally extracted information such as retinal blood vessels and vessel diameter [31, 32]. Nathaniel M. Braman et al. applied Gabor wavelet in both intratumoral and peritumoral regions to extract detailed edge information [11]. The Gabor wavelet features based on manually selected regions tend to show an unreliable performance as manual selection leads to loss of tumor shape information due to inter-observer variability.

Despite the findings presented herein, this study had three major limitations. Firstly, the retrospective nature of the study lacks external validation outside a single institution. Secondly, sampling bias may exist as a result of the small sample size in our study. The small sample size was caused by the strict inclusion and exclusion criteria. Therefore, the results in the present investigation also need to be verified by further studies. The third limitation is the unbalanced sample sizes of the experimental group and the control group. Although a SMOTE algorithm was used to balance the data, some bias may still exist between the two groups.

## Conclusions

Our study demonstrated that wavelet-transformed textures outperformed intratumoral and peritumoral textures for radiomic MRI prediction of pCR to NAC for patients with LABC. Therefore, the method presented in this study may provide a potential surrogate for the accurate prediction of the clinical outcomes of NAC, resulting more effective treatment.

## Supplementary information

**Additional file 1 Table S1.** Comparison between our results and the recent obtained results.

## Data Availability

The data that support the findings of this study are available from the corresponding author upon reasonable requests.

## Abbreviations

AUC: Area under curve
CE-MRI: Contrast-enhanced magnetic resonance imaging
NAC: Neoadjuvant chemotherapy
pCR: Pathological complete response
ROC: Receiver operating characteristic
VOI: Volume of interest
