# Supplementary material for: Predicting the response to neoadjuvant chemotherapy for breast cancer: wavelet transforming radiomics in MRI
Source: BMC Cancer. 2020 Feb 5;20:100. doi: 10.1186/s12885-020-6523-2 (PMC7003343; doi:10.1186/s12885-020-6523-2)
Supplement: Supplementary file 1 — Additional file 1 Table S1. Comparison between our results and the recent obtained results. [file 12885_2020_6523_MOESM1_ESM.docx]

| **Study** | **Year** | **Method** | **Sample size** | **MRI sequence** | **NAC period** | **Response/**  **Non-response** | **AUC** | **Accuracy** | **Sensitivity** | **Specificity** |
| --- | --- | --- | --- | --- | --- | --- | --- | --- | --- | --- |
| Mani S et al. | 2013 | Machine learning | 28 | CE and DWI | Before and after one cycle MRI | 11/17 | 0.860 | 0.860 | 0.880 | 0.820 |
| Cain EH et al. | 2019 | Machine learning | 288 | DCE-MRI | Pre-treatment MRI | 64/224 | 0.707 | / | / | / |
| Our study | / | Machine learning | 55 | CE-MRI | Pre-treatment MRI | 17/38 | 0.888 | 0.810 | 0.762 | 0.845 |

**Supplement Table. Comparison between our results and the recent obtained results**

Abbreviations: AUC, area under the curve; NAC, neoadjuvant chemotherapy.
